# Secure information transport by transverse localization of light

**DOI:** 10.1038/srep29918

**Published:** 2016-07-20

**Authors:** Marco Leonetti, Salman Karbasi, Arash Mafi, Eugenio DelRe, Claudio Conti

**Affiliations:** 1 Center for Life Nano Science@Sapienza, Istituto Italiano di Tecnologia, Viale Regina Elena, 291 00161 Roma (RM) Italia; 2 Department of Electrical and Computer Engineering, University of California, San Diego, La Jolla, CA 92093, USA; 3 Department of Physics and Astronomy and Center for High Technology Materials, University of New Mexico, Albuquerque, NM 87131, USA; 4 Dep. Physics University Sapienza, P.le Aldo Moro 5, 00185, Roma Italy; 5 ISC-CNR and Department of Physics, University Sapienza, P.le Aldo Moro 5, I-00185 Roma, Italy

## Abstract

A single-photon beating with itself can produce even the most elaborate optical fringe pattern. However, the large amount of information enclosed in such a pattern is typically inaccessible, since the complete distribution can be visualized only after many detections. In fact this limitation is only true for delocalized patterns. Here we demonstrate how reconfigurable localized optical patterns allow to encode up to 6 bits of information in disorder-induced high transmission channels, even using a small number of photon counts. We developed a quantum key distribution scheme for fiber communication in which high information capacity is achieved through position and momentum complementarity.

The hallmark of quantum optics is a single-photon in a double-slit experiment: the uncertainty in the path of the particle gives rise to a generally elaborate fringe pattern. Although this pattern is caused by the photon beating with itself, it is spatially extended and materializes only after many single photon detections. In order for the pattern to emerge after just one detection, it must be strongly localized. Apart form standard localization schemes such as the focal point of a lens, a more tunable and versatile form of localization is supported in disordered media. Recent experiments^1^,^2^,^3^ have in fact shown how disorder can lead to unexpected quantum and classical optical interference phenomena, such as the ability to focus light through a disordered material^4^,^5^,^6^ or transversally localize light into a disordered optical fiber^7^,^8^. Specifically, photons are the elementary excitations of the modes of light. In a laser or an optical fiber, these modes are easily recognizable and classified. By contrast, following a photon in a disordered system through a patchwork of states is generally meaningless: the field spreads out forming disorganized speckles. In turn, disorder can itself cause strong spatial localization^9^,^10^,^11^,^12^,^13^ while an active modulation of the input wavefront enables adaptive focusing^14^. In distinction to standard localization, such as when a laser beam is focused down to a spot using a lens, localized light in a disordered medium only occurs for very precise and elaborate input waveforms.

The actual focusing input solution depends both on the specific disorder encountered and on the position in which the spot is formed. The result is that localized light can potentially carry large amounts of information because the number of possible fringe patterns caused by the interplay of the wavefront with disorder is large. Since the effect is interferometric, we expect it to be equally true down to the one-photon-at-a-time level, i.e. to be detectable for a very limited number of photon counts. In this case each single photon at the output spot is heavily encoded by the disorder/wavefront interplay.

Here we use disorder and reconfigurable input wavefronts to encode ultra-weak laser beams with up to 6 bits of information per photon detection. The proposed scheme, allows for quantum key distribution of this information exploiting momentum and position complementarity.

Our finding hinges on the mapping of input photon waveforms onto a grid of highly transmissive localized channels in a disordered fiber. To obtain localization with an ultra-weak beam to form an encoded transmission, we combine adaptive optics based on a spatial light modulator (SLM) with Anderson localized (AL) states in a binary random plastic optical fiber. Since a random material does not generally guide, we use AL to allow the adaptive optics focalization to work through the entire length of the fiber, that is, in conditions in which the input waveform is launched at one port and the adapted focus forms at the other. AL is common to all kinds of waves, ranging from matter waves^15^,^16^ to one-dimensional quantum walks^17^,^18^. While AL in three dimensions (3D) is a challenge^19^,^20^,^21^,^22^,^23^ here we exploit two-dimensional (2D) *transverse*^24^ localization, which is always obtained in sufficiently large samples with disorder in the plane perpendicular to the direction of propagation^7^,^24^,^25^,^26^. Localized states have never been exploited for quantum key distribution so far. Quantum key distribution allows the sifting of a secure common key between two parties A and B with unconditional security using quantum effects^27^,^28^,^29^,^30^. In the basic BB84 protocol, single two-level systems are first encoded by A onto two noncommuting bases chosen at random, sent to B, and decoded using the encoding bases but now chosen at random by B. On declaring the bases chosen, A and B can then sift a secret common key formed by those events in which the encoding and decoding were carried out in the same basis. Security can be checked by comparing a sub-set of common valid key values, since any intereference on behalf of an eavesdropper E will inevitably have a finite probability of changing the encoded bit and hence causing detectable errors, alerting A and B as to the intrusion. The protocol can be generalized in many ways, for example from discrete to continuous variables. At present, great attention is given to secure key distribution using single photons and optical fiber. Here the system of choice is the use of polarization states as encoding and decoding bases, the noncommuting bases being for example linear and circular polarization. Even considering attenuation, birefringence, and dispersion, this forms a pragmatic setting, since single mode fibers can be used and detection can be carried out without the use of interferometers. In truth, each single photon can carry far more information than a single polarization qubit, the limit to the amount of information being fundamentally constrained by noise. We here explore and demonstrate how a disordered fiber can be used to enact a multi-bit quantum key distribution optical fiber scheme.

## Materials and Methods

**Figure 1:**
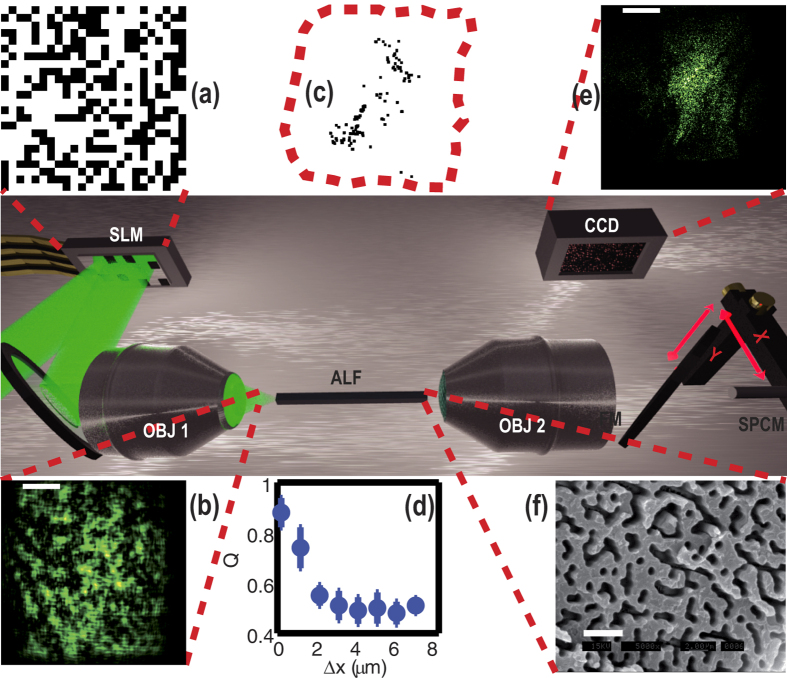
Experimental setup. In the insets: (**a**) SLM input mask; (**b**) laser spot at the ALF input, scale bar is 10 *μ*m; (**c**) black squares indicate the location of the high transmission channels at the fiber output (fibers boundary are the red dashed line); (**d**) we report the degree of matrix similitude Q as a function of the displacement Δ*x*; (**e**) laser at the fiber output with the input shown in (**b**); scale bar is 40 *μ*m (**f**) disordered fiber, dark areas correspond to PMMA, the rest to PS; Scale bar is 4 *μ*m.

Our Anderson localization fiber, (ALF), (Fig. 1f position of high transmission channels in panel c) has been fabricated by melding 40,000 strands of polystyrene (PS) and 40,000 strands of polymethyl methacrylate (PMMA)^31^. The mixture of strands was fused together and redrawn to a square shaped fiber with a lateral size *S* = 250 *μ*m and a length of 8 cm^31^, with a random distribution of the two materials having a refractive index mismatch of 0.1. The resulting localization length (of the order of micron), is much smaller than the fiber size^10^,^11^. Light generated by a continuous-wave (CW) laser with wavelength *λ* = 532 nm is injected in the system by a long working distance (OBJ 1 in Fig. 1) objective. We use two input configurations: in configuration I) a collimated beam is injected on the objective to obtain a focused spot size of 0.7 *μm*; in configuration II) a lens is placed before the objective to create a de-magnified image of the Spatial Light Modulator (SLM) on the input face of the fiber. A collection objective (OBJ 2 in Fig. 1) creates an enlarged image (×28) of the ALF output, on a plane where a 50 *μ*m core collection optical fiber resides, bringing the light to a single-photon-counting module (SPCM) or to a standard photodetector. The collection fiber scans the image plane by means of two computer-controlled precision motors. A flip mirror (FM) is used to obtain an image of the ALF output on a CCD (Fig. 1e reports an image of the output of the ALF).

## Result and Discussion

**Figure 2:**
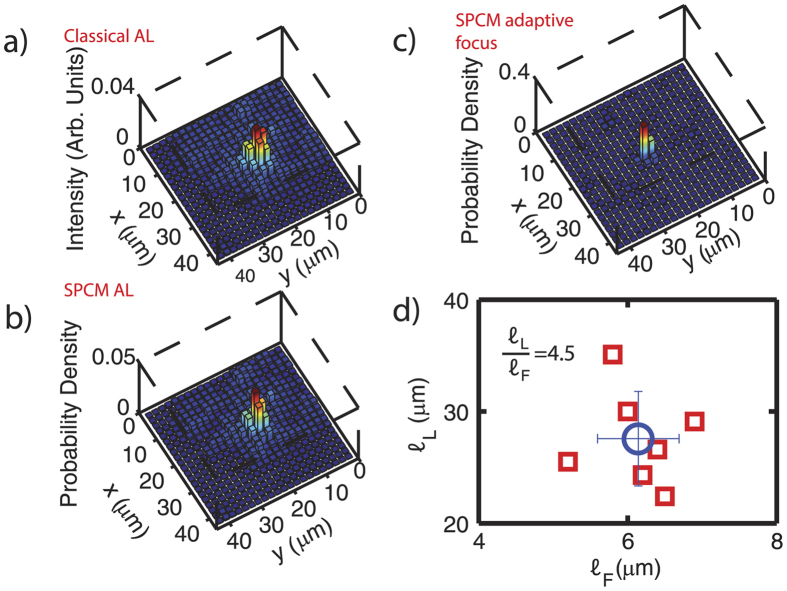
(**a**) Classical AL. Intensity measured by a scanning fiber (configuration I); (**b**) Photon probability density measured with scanning fiber and SPCM (configuration I); (**c**) One-photon-at-a-time adaptive focusing obtained after the optimization procedure (configuration II); (**d**) Localization length of the mode 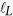 plotted versus the localization length after the optimization procedure 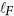 retrieved for 6 different modes. Error on the individual measurement is smaller than the marker size. The open circle with error bars represents the average over all the investigated localizations.

Our Anderson-based encoding and encryption system relies on a quantum key distribution algorithm similar to the one used in commercial quantum cryptography, i.e., based on standard laser sources highly attenuated in order to improve the single photon component. In Fig. 2 we measure the shape of a single localized state in the low light regime with a single photon counting module. Tuning the entrance position in the ALF (in the input configuration I), light is coupled to a localized mode of the AL fiber, thus light remains confined up to the fiber end. The output intensity is retrieved with a standard detector and reported in Fig. 2a in which the bar height is proportional to the intensity measured at the corresponding X-Y position. We next strongly attenuate the laser beam and monitor transmission using an SPCM (see Supplementary Information), in conditions in which at most a single detection event occurs per gate with an approach similar to that exploited for commercial quantum key distribution^32^,^33^. In Fig. 2b we report the probability density to detect an event measured scanning the collection fiber in the low light regime. As theoretically expected, the single photon probability density is a replica of the intensity map and consists of a central peak of 0.04 with full width at half maximum FWHM = 6 *μm* towering on a speckled background.

We next proceed to demonstrate adaptive optics focusing through the ALF in the low light regime. To this aim we exploit the configuration II) in which the SLM is imaged onto the ALF input. This allows us to localize light down to 2 *μm* as shown in Fig. 2c 8, see Supplementary Information. Compared to the previously reported less-localized AL states, this adaptive focusing is based on the idea that a light beam passing through a disordered medium forms a speckle pattern originated from the random phase delay distribution: compensating this phase delay through an SLM produces a focus at a user-defined target position^4^,^5^,^6^.

Compensation is obtained through an optimization procedure that exploits a feedback mechanism similar to that of ref. 6: the SLM is divided in 24 × 24 segments which may be individually turned “on” or “off”, as shown by Fig. 1a in which black segments impress a *π* phase delay on the reflected beam. In our case the feedback is obtained by the SPCM, and the position at which the intensity/probability density is enhanced (the “target” of the optimization) is defined by the collection fiber position (see Supplementary Information).

The scan of the probability density corresponding to an optimal input matrix is reported on Fig. 2c. The peak is in the same position of the AL of Fig. 2b and reaches a maximum of 0.25 with a of FWHM = 2 *μm*. More than 80% of the probability density is found in a square of 15 *μ*m side centered at the maximum. To characterize the degree of localization we exploit the localization length 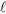 obtained from the inverse participation ratio 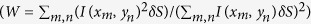 (which has the unit of an inverse area, and where Δ*S* is the size of the surface element) as 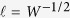. The single mode localization length, 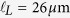 (Fig. 2a,b), decreases to 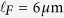 when multimode activation is exploited (Fig. 2c) through the optimization procedure. A similar result is found for all the investigated localized states. In Fig. 2d we report the localization length of several localized modes 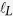 plotted as a function of the localization length 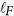 obtained at the same location exploiting the adaptive focusing. Adaptive focusing allows a degree of localization higher than that provided by the fiber modes: 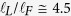. The same ratio is >100 for a homogeneous fiber^8^.

**Figure 3:**
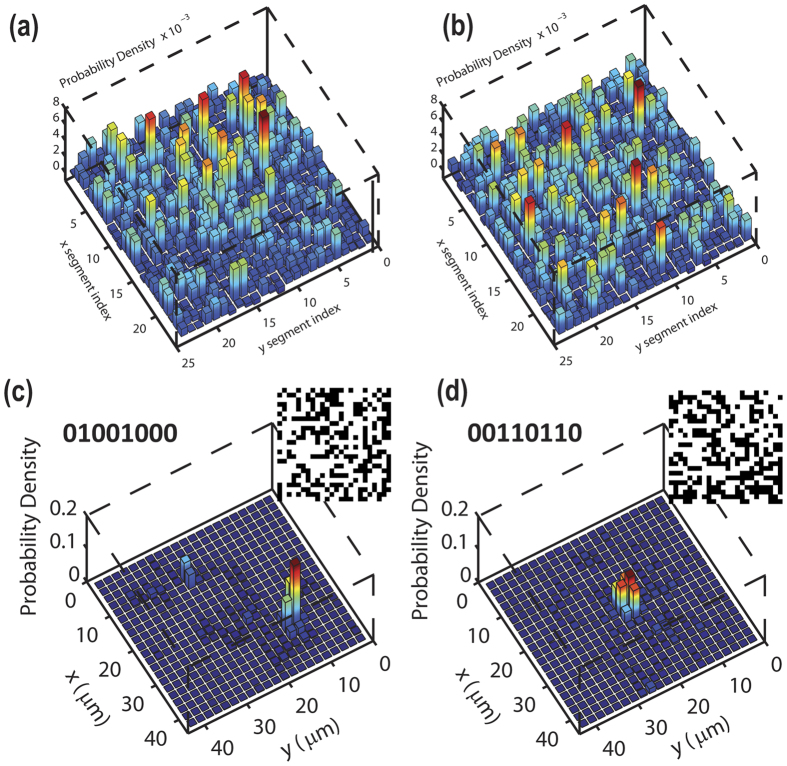
(**a**,**b**) Contribution of each of the SLM segments to the probability density at the peak. The two patterns are relative to the optimization of panels 3(c,d) respectively. (**c**,**d**) probability density at the fiber output obtained with two different optimization targets. The amplitude masks of the SLM are reported in the insets.

That adaptive focus is obtained by an extended input. This is demonstrated by Fig. 3a,b in which we show the contribution W(i, j) of every SLM segment (i, j) on the total intensity of the focus (see Supplementary Information). Since W(i, j) has an high value over many segments, the input is delocalized.

In Fig. 3c,d we demonstrate that we are able to scan an arbitrary position (x, y) in the output plane changing the optimization target. Distinguishable output spots correspond to uncorrelated input matrices (see insets in Fig. 3c,d which represent the input SLM mask). The degree of similitude between two matrices M and N is quantified by the fraction of identical values Q(M, N). Figure 1d shows Q for SLM matrices that optimize localized spots Δ*x* apart demonstrating that foci distant more than 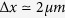 are generated by statistically uncorrelated input (Q = 0.5).

Now we will exploit the localized states as information channels. Indeed, the AL states in our fibers form a sparse and disordered distribution of transmission channels; this configuration is optimal for transmitting information because the detection of an event in correspondence of a particular output indicates that the transmitter is using the corresponding channel to transmit. The information may be then carried from one side to the other of the fiber labeling any channel with a symbol, number or letter. The selection of localized modes which are exploited for the communication is a critical point of our communication protocol. Each mode is characterized by a specific probability density function, located at a certain position at the fiber output. Increasing the number of modes which are exploited for communication, increases the amount of information which is encoded in a single photon, but also increases also the error probability, a photon may fall into a neighbor channel resulting in a wrong communication. We estimate that the fiber under examination posses at least 4000 localized states which may be selectively activated with the SLM. Exploiting all these modes would result in a low average efficiency and large misdetection probability (see below). Here we set arbitrarily a minimum requirement of 80% efficiency (80% of the transmitted light is brought at the target, see Supplementary Information) in order to ensure an efficient transmission.

Figure 1c shows the arrangement of 92 the highly transmitting channels meeting the requirements. As an example, by decreasing the requested efficiency to 70%, 142 modes meeting the requirements have been found. We found a comparable amount of high throughput channels on each disordered fiber (with different disorder realization) we analyzed. Exploiting less efficient channels would reduce the effectiveness of our protocol. By excluding localizations that are too close to one another (less than 15 *μm* distance), we obtain *NS*_*X*_ = 77 highly efficient and sparse transport channels which are individually addressable with the SLM (exploiting the proper input matrix): single photon counts corresponding to light mapped one to one of these output states carries 6 bits of information (6 bits = 64 available states). Eventually, a larger number of bits can be encoded at expenses of efficiency by exploiting weaker channels. Selectively inserting a lens at the output allows also to span both position X ≡ (x, y) and wavevector K ≡ (*K*_*x*_, *K*_*y*_) space^34^. The number of efficient (more than 80% of transmission) states found in the K space is *NS*_*K*_ = 72 (also in momentum space 6 bits of information are available).

**Figure 4:**
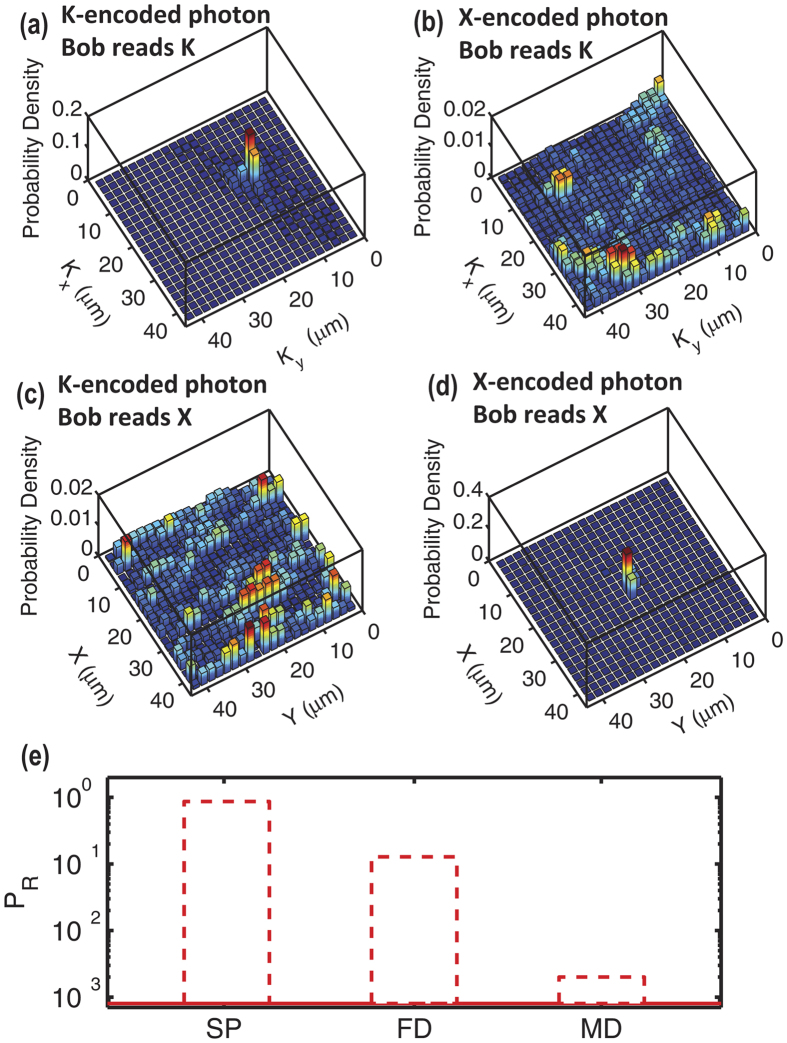
In (**a**) localization is preserved when A encodes K and B reads K. In (**b**) localization and the encoded information is lost when A and B use mutually incompatible bases X and K. In (**e**) detection probability of the message 72, (the positions space.) with relative value of failed detections (FD) and misdetections (MD). Note the Y log scale.

We next apply to our communication scheme a multi-bit BB84 quantum cryptography protocol^34^. Quantum cryptography is based on the transmission of single q-bits from Alice (A) to Bob (B) on mutually incompatible basis, i.e., *S*_*x*_ and *S*_*y*_ for an electron. The same scheme can be reproduced for the position *X* and wavevector *K* of the photon. A/B encode/readout *X* or *K* adding an imaging lens, performing a spatial Fourier transform in the collection arm of their experiment. A has a set of optimized SLM matrices that lead to *NS* = 149 distinguishable high transmission channels. She now selects a specific SLM matrix and sends the field to B. B launches it into the fiber (that can either be given to B by A or is public), and B decides to detect *X* or *K*. Hence, as described in ref. 34, A encodes a photon in *X* or *K*, and B reads it in *X* or *K*. After the publication of the chosen basis, valid keys received by B are sifted and security checked. The principle of operation is shown in Fig. 4a,b. In Fig. 4a A encodes *K* and B decodes in *K*, so the original selected byte is transmitted with a localized distribution. In Fig. 4b A encodes *X* but B detects in *K* so that the original information is lost (resulting in a speckle pattern). If A encodes in *X* while B encodes *K* again a disorganized speckle is found (Fig. 4c), while if both encodes in *X* a localized state is retrieved (Fig. 4d).

The feasibility of the technique to encode messages is confirmed by Fig. 4c. The first column on the left represent the probability to successfully detect the message “72” (state arbitrarily chosen) and is close to 90% (see Supplementary Information). There is a 10% of failed detections (black bar, see Supplementary information) while the percentage of misdetections (photons detected in an area pertaining to a channel different from the target, see Supplementary information) is of the 1%. QKD protocol needs a two set of redundant states so that the same message may be either dispatched on K or X. This means that if one wants to exploit QKD in our ALF has to limit the number of bits per photon to 6: 2^6^ = 64 states, <*NS*_*X*_ + *NS*_*K*_.

Our scheme is based on the fact that a single-photon is able to bear all the information contained in an interferogram and deliver it through a single photon detection using strong localization. As in commercial QKD systems with decoy state protocols, we use an attenuated laser beam^32^,^33^. In these conditions, heralded photons increase the detection rate, but this at the cost of added complexity^35^,^36^.

## Conclusions

In summary, we combined localized states and adaptive focusing with a quantum key distribution system based on attenuated coherent laser beam. Light, from a spatially extended input, can be arbitrarily reconfigured by actively selecting the target of an optimization procedure to be focused into one in a constellation of high transmission channels. The sparse and localized nature of the channels allows to build an efficient, high capacity, quantum key distribution system. Adaptively optimized transport through localized states ensures that 80% of light is focused at the target. On the other hand the sparsity of the channels reduces the misdetection probability (1%). We realized a spatial coding in which a single detection is encoded with up to 6 bits of data while, exploiting quantum encryption with position and momentum as complementary variables, the transmission is quantum-secure.

## Additional Information

**How to cite this article**: Leonetti, M. *et al*. Secure information transport by transverse localization of light. *Sci. Rep.* **6**, 29918; doi: 10.1038/srep29918 (2016).

## Supplementary Material

Supplementary Information
