# Supplementary material for: Secure information transport by transverse localization of light
Source: Sci Rep. 2016 Jul 20;6:29918. doi: 10.1038/srep29918 (PMC4951778; doi:10.1038/srep29918)
Supplement: Supplementary Information [file srep29918-s1.pdf]

Secure information transport by transverse localization of light

Marco Leonetti,[1] Salman Karbasi,[2] Arash Mafi,[3] Eugenio DelRe,[4] and Claudio Conti[5]

[1] *Center for Life Nano Science@Sapienza, Istituto Italiano di Tecnologia, Viale Regina Elena, 291 00161 Roma (RM) Italia**

[2] *Department of Electrical and Computer Engineering, University of California, San Diego, La Jolla, CA 92093, USA*

[3] *Department of Physics and Astronomy and Center for High Technology Materials, University of New Mexico, Albuquerque, NM 87131, USA*

[4] *Dep. Physics University Sapienza, P.le Aldo Moro 5, 00185, Roma Italy*

[5] *ISC-CNR and Department of Physics, University Sapienza, P.le Aldo Moro 5, I-00185 Roma, Italy*

(Dated: March 3, 2016)

METHODS

Measurement protocol Light is injected into the fiber through a 1 mW beam attenuated using a neutral reflective filter with optical density 4. Photons are detected on average once every ten gates using a 10^{-5} s integration window for the single photon counting module. Measurements are performed respecting the 0.1 counts per gate upper bound in order to comply with quantum key distribution requirements: two photon events are limited to less than 5% of the clicks.

Attenuation The ALF presents two kinds of disorder: transverse disorder and longitudinal disorder. Transverse disorder is deliberately introduced at the fabrication stage and is the responsible for the transverse localization. Another contribution to disorder is longitudinal (unwanted) disorder which is introduced due to fabrication imperfection. The causes of this longitudinal disorder which reduces the total transmission of the fiber are two :dirt introduced at the assembly stage and residual humidity which is instead included at the fiber drawing stage. At present, these two contributions introduce an attenuation of 1dB per centimeter, making the ALF transmission feasible with our sample only for short range communication. On the other hand, assembling the fiber in clean environment and reducing the water content the attenuation may be reduced by a factor 10^4, which together with the decoy state technology[1, 2] can support ALF based medium range communication.

The optimization procedure is as follows: A) A reduced size image of the SLM in the amplitude-only configuration is projected on the fiber. B) A set of 100 random input masks are imaged on the SLM producing a random speckle pattern (Fig. 1b) at the input. The mask producing the highest peak count rate (acquisition time is set to 1 second) is chosen to start the next part of the optimization protocol; C) One segment of the SLM is flipped and the change is retained if the number of detected photons is increased; D) Step C is repeated for every segment of the SLM, to obtain at the end of the protocol an "optimal input matrix".

W(i,j) is measured at the end of the optimization procedure: starting from the SLM "optimal matrix", we switch the transmission state of the segment (i,j) and we measure the decrease of the probability density W(i,j) at the "target" (the position at which the peak forms). In Fig. 3 we show W(i,j) for each of the 24×24 SLM channels. The two distributions correspond to two different targets (Fig. 3a is relative to Fig.3c and Fig. 3b is relative to

Fig. 3d).

Labeled states Information is encoded through the ALF channels by labeling the states. In our protocol the states are labeled starting from the upper left of the image 4b with growing natural numbers. A critical parameter to recognize the information is the area of the output fiber associated with a state. We defined an area corresponding to a labeling square with 15 μm side centered on the position of the channel maximum intensity. As an example, in the case of the photons encoded in the channel of figure 3c, if a photon falls in the labeling square centered at the coordinates (30 10) it is recognized as the message "72".

Communication scheme and efficiency measurement For a successful communication, the receiver must be able to identify the localized state which has been chosen by the transmitter . A state is identified if a photon detection is obtained in a pre-determined area of the fiber output tip defined in agreement between the transmitter and the receiver: the detection of a photon in a specific area which is "served" by a localized state, corresponds to a specific message. We associate to every channel a "letter" so that, after several detections, i.e., several letters collected, the receiver is able to reconstruct the full key. The transmitter (Alice) aims to gather all (almost all) light in the corresponding state, so that when the receiver (Bob) detects a photon it will be located at the target state and the "letter" may be identified without errors. If a mode is not sufficiently localized then error may occur because it may contribute to areas labeled with different letters (photons encoded to deliver the letter "A" which instead deliver letter "B"). Errors are caused by photons which are encoded into a target state but detected into a different one due to the residual probability density which is not strictly zero out of the area corresponding to the target mode. This residual probability density causes increased error count rates (the missdetection rate, MD) and may jeopardize the QKD success. By choosing, for labeling, only modes that are far away, the MD can be reduced.

Photons falling in an area which is not labeled, are counted as a Failed Detections (FD). The value of FD is critical because variations of FD will detect the presence of an eavesdropper. The success probability, missdetection probability and failure probability vary from state to state, due to the random distribution of localized states, which may lie close or far from each other (if a state is far away from the neighbors the missdetection probability is lower). In the experiment reported here we found that our fibers posses about 4000 localized states. We selected to be labeled, only modes producing an SP higher than 80% and an

MD lower that 1%: in the example reported in the main text, this selection leads 149 states meeting the requirements.

To address the success probability of a state, we encode at the input in the chosen channel ("letter") by exploiting a corresponding SLM mask and the right basis (in both transmission and detection). In our measurement protocol, we set the total exposure time to 1s (10^5 gates) for each pixel (bars in Fig. 4(a-d)). Then we count as successful detections (whose occurrence probability is indicated as SP in graph 4e) the photons falling in the area of the fiber corresponding to the labeled states. The photons detected in an area which pertains to no localization are failed detections (FD on the graph 4e), while we count as a misdetection (indicated in the MD bar of figure 4e) the photons falling into an area labeled with a state different from the encoded one.

Below we report two figures containing the graphs (left column of graphs) reporting SP, FD, and MD probability for the first 10 modes of our mode set (mode number reported in the graph's title). The insets on the right report a scheme of the fiber output (fiber side is 250 microns) in which black squares represent the modes' positions. The blue circle indicates the position of the modes whose characteristics are reported on the graph on the left. All the other localizations (states labeled from 11 to 149, not reported), show an SP higher than 80% and a MD lower than 1%, as we selected only modes fulfilling the communication requirements.

A sketch of the communication scheme is reported in Fig. 3. Alice encodes photons at a pre chosen position in K or X space by exploiting masks which have been previously retrieved and stored into a database. On the other side of the fiber Bob chooses to read in K or X by exploiting a flip mirror which. On one arm there is a lens which builds an image of the fiber output tip (X space), on the other there is instead the K space.

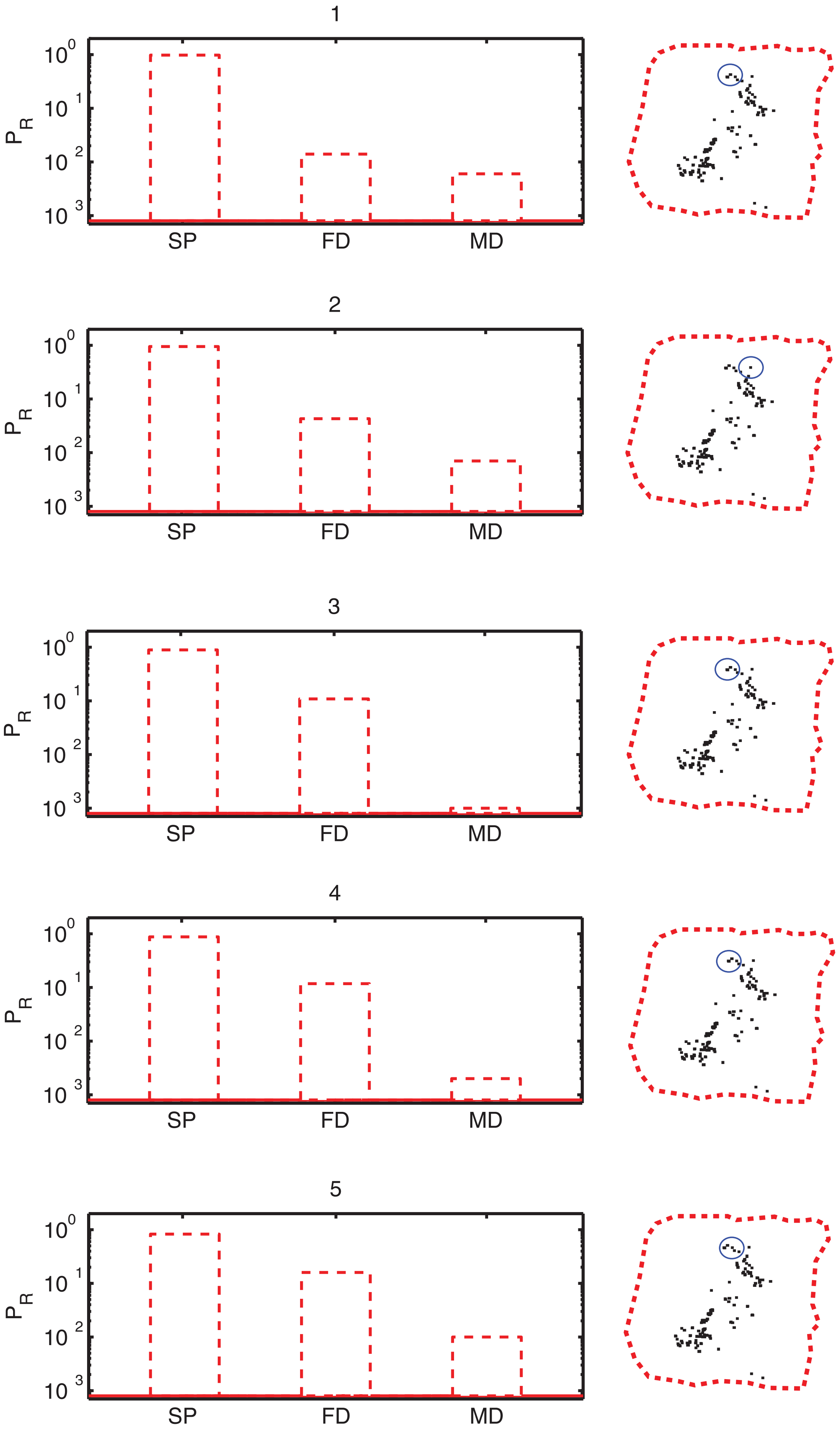

FIG. 1. Success probability (SP), failed detections (FD), and missdetections (MD) for the first five modes are indicated in the left bar graphs. Blue circle in the inset on the right represent the mode location with respect to the fiber output facet (boundaries indicated in red).

* Corresponding Author: marco.leonetti@roma1.infn.it

[1] H.-K. Lo, X. Ma, and K. Chen, Phys. Rev. Lett. **94**, 230504 (2005).

[2] A. R. Dixon, Z. L. Yuan, J. F. Dynes, A. W. Sharpe, and A. J. Shields, Opt. Express **16**, 18790 (2008).

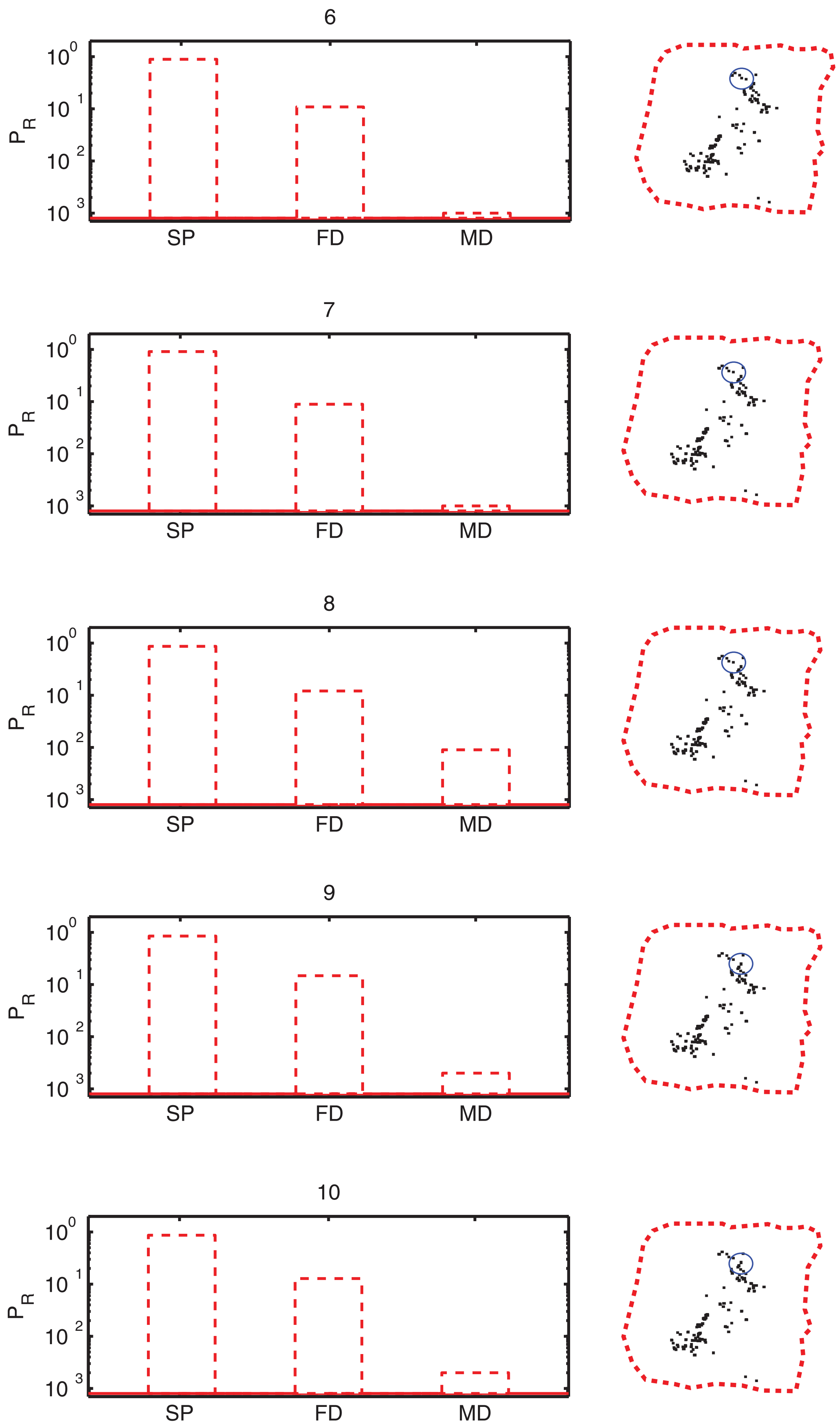

FIG. 2. Success probability (SP), failed detections (FD), and missdetections (MD) for modes 6-10 are indicated in the left bar graphs. Blue circle in the inset on the right represent the mode location with respect to the fiber output facet (boundaries indicated in red).

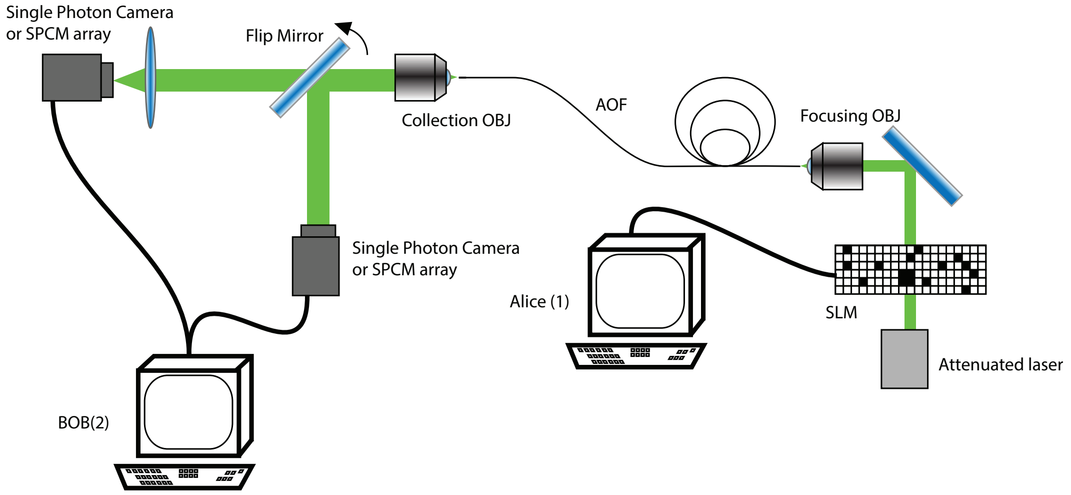

FIG. 3. Scheme for quantum communication through Anderson fibers.
